# Clinical mental health supervision in a humanitarian context in LMICs: PEACE model for community engagement

**DOI:** 10.1186/s40900-025-00722-2

**Published:** 2025-10-10

**Authors:** Sabiha Jahan, Md. Omar Faruk, Gülşah Kurt, Shaun Némorin, Ariel Zarate, Muhammad Kamruzzaman Mozumder, Simon Rosenbaum, Ruth Wells

**Affiliations:** 1 Department of Clinical Psychology, University of Dhaka, Dhaka, Bangladesh; 2 Service for the Treatment of Torture and Trauma Survivors, Sydney, Australia; 3 School of Psychology, University of New South Wales, Sydney, Australia; 4 School of Psychiatry, Faculty of Medicine, University of New South Wales, Sydney, Australia; 5 Discipline of Psychiatry and Mental Health, School of Clinical Medicine, University of New South Wales, Sydney, Australia

**Keywords:** Clinical supervision, Community voice, Community engagement, Humanitarian contexts, Mental health

## Abstract

**Background:**

The Caring for Carers (C4C) project aims to assess the effectiveness and acceptability of an online, group-based supervision program for mental health practitioners working with displaced communities in Bangladesh and in Türkiye and Northwest Syria. This paper highlights the integration of Rohingya perspectives to ensure responsiveness to the unique needs of displaced populations through the supervision program.

**Methods:**

Adopting a community-based participatory research (CBPR) approach, the project engaged Rohingya community members in every phase to ensure the program’s relevance to local needs. A Rohingya Advisory Committee (RAC) was formed to integrate Rohingya perspectives and conducted in-depth interviews (IDI) and focus group discussions (FGD) with two female MHPSS service users and five male community members, respectively. As recommended by Kiger and Varpio (2020), thematic analysis was employed within a constructivist framework that acknowledged cultural variations in mental health perceptions. The C4C project employed several strategies to engage community members and integrate their perspectives. Stakeholder consultations involved MHPSS service users and community members, the RAC conducted workshops for supervisors, and engaged regularly with the project team providing insights on cultural and practical challenges and collaborating to adapt supervision program materials.

**Results:**

FGD and IDI with service users and community members provided a first glimpse into the community’s needs, and experiences, whereas the advisory committee provided lived experiences, meaning of displacement, and ancestral background. The project team invested in a respectful relationship with the advisory committee, working collaboratively to reflect on each other’s perspectives through regular meetings and adding content and strategies to the supervision processes. Voices from the community informed the supervision program by elucidating contextual markers, cultural and situational understanding, appreciation, curiosity, experiential information, historical background, and perspectives on mental health needs as well as services.

**Conclusions:**

Incorporating strategies and perspectives from the community, we aimed to provide a framework of community engagement termed as PEACE (Participation, Expertise, Agency, Connection, and Empowerment) along with enabling and challenging factors.

**Supplementary Information:**

The online version contains supplementary material available at 10.1186/s40900-025-00722-2.

## Abstract (plain language summary)

Involving mental health service users and community members for which the services have been developed is a crucial stage to ensure mental health services are culturally sensitive. This is even more important for those representing forcefully displaced communities amid limited or no opportunities to contribute to the development and delivery of these services. Often, the voices of these communities are less sought raising concerns over the suitability and acceptability of the services. Community-based participatory initiatives help to include the voices of refugee or displaced communities in research and clinical practice. We aimed to collaborate with with Rohingya refugee community members living in Cox’s Bazaar, Bangladesh to ensure mental health services adequately capture their needs. A Rohingya Advisory Community (RAC) was formed in an effort to understand their needs and opinions to be able to provide culturally sensitive mental health services. Researchers initiated and continued discussions with the RAC for over 2 years. The RAC engaged in several activities such as workshops and presentations to help design a program to support Bangladeshi mental health practitioners to provide mental health care taking the cultural into account.

Based on the discussion, we aimed to share a framework- PEACE, to guide meaningful participation in low-and-middle income countries. PEACE stands for **Participation**, respecting community **Expertise**, promoting **Agency**, building genuine **Connections**, towards **Empowerment**. In addition, we also discussed potential challenges such as lack of trust and resources, human rights violations to foster meaningful participation. Finally, we emphasized that models of participation from Western countries must be guided by the knowledge of community people who have better cultural, social, and political understanding.

**Supplementary Information:**

The online version contains supplementary material available at 10.1186/s40900-025-00722-2.

## Introduction

Engaging displaced communities in humanitarian responses can promote culturally sensitive approaches that can improve the quality and accessibility of services and thereby increase the sustainability of services [1]. Community engagement in humanitarian crises has gained an increasing priority [2] with major international donors that, in 2016, pledged to allocate 25% of humanitarian funding to locally run initiatives to boost meaningful participation of displaced communities in the health system programming that affects them [3]. However, there is a long way to go to put participatory methods into practice in humanitarian settings [4]. A recent study showed that less than 2% of funding goes to locally run organisations in low- and middle-income countries (LMICs), where 90% of the world’s 110 million displaced people reside [5]. Strengthening community engagement and directing more resources to local organizations can enhance sustainable and effective support for displaced populations. Most research with displaced communities focusing on participatory methods is conducted in high-income countries (HICs) [6]. Comparatively little research has examined the specific processes needed to conduct participatory research in LMICs or humanitarian settings [4]. In the present study, we describe the process of engaging in collaborative dialogue throughout a 3-year community-based participatory research (CBPR) project aiming to improve mental health services for Rohingya people displaced in Bangladesh. The project brought together researchers from a LMIC university (University of Dhaka, Bangladesh), members of the Rohingya refugee community displaced in Bangladesh and a HIC university (UNSW Sydney, Australia). We argue that through collaborative and responsive relationships, trust can underlie humanitarian health systems that respect the sovereignty of displaced communities.

The engagement of affected populations in humanitarian responses can enhance community trust while sensitively addressing pressing community priorities [7]. This also helps to ensure interventions are effective and sustainable [8, 9] which in turn affects meaningful participation [10]. Rohingya community members in Bangladesh can offer knowledge on the culture, customs, and pertinent contextual factors within the humanitarian context. These insights can help ensure mental health services are culturally sensitive and contextually relevant which will likely increase acceptability and effectiveness of mental health and psychosocial support (MHPSS) services [11]. Additionally, integration of community voices can help ensure community engagement and ownership of MHPSS service provision.

Our current study builds on the premise that community engagement needs to be utilized as a transformational process [12] starting from the inception of research to the implementation of results. This is easier said than done. Most commonly, participation in global health is implemented by community health workers engaged to improve effectiveness by boosting contextual and cultural appropriateness and awareness in health services [13]. Globally, the mental health service user movements has significantly increased community involvement in research and programming [14]. In humanitarian settings of LMICs, organizations often consult communities, but it is less common for programs to engage community members in decision making, either through advisory committees or by supporting finance and infrastructure towards local-run initiatives [6]. The mere use of community engagement as an instrument to collect data and obtain feedback on interventions has been criticized for failing to transform the power dynamics inherent in global health research [12].

Given that most research on CPBR has been conducted in HICs [6], it is important to consider the unique challenges, risks, and opportunities for engaging community voices in research in humanitarian settings in LMICs [15]. A 2020 analysis of participatory research in humanitarian settings identified trust building and understanding the dynamics of the crisis setting as crucial to successful community engagement. In particular, allowing time for trust to build, remunerating people for their time, setting up small groups, and confidential opportunities were key to enabling community members to safely and openly share their ideas and experiences [4]. In such complex settings, with displaced communities facing so many sources of exclusion and disempowerment, it is not our aim to present a ‘success’ story. No single research process can overcome the structural inequalities produced within the global health research system. In this system, epistemic injustices favour English speakers from HICs [16] and undermine the credibility of people from refugee communities as knowers of their own realities [17]. We aim to reflect on our process and the power dynamics that can influence implementing CBPR in humanitarian contexts in LMICs.

The Caring for Carers (C4C) project aims to test the impact and acceptability of an online group-based supervision program for mental health workers providing support to Rohingya displaced communities in Bangladesh, and Syrian displaced people in Türkiye and Northwest Syria. Mental health workers in humanitarian settings experience significant stress due to their exposure to vicarious trauma, prolonged working hours, and volatile working environment [18–21] all of which increase their risk of experiencing mental health problems [20]. Research has shown that supervision can increase practitioners’ promotion and adherence to quality health care [see 22], it ameliorates mental health burden, and increases therapeutic alliance among recipients [23–25]. We aimed to create a collaborative virtual space where trust could be built across cultures and contexts and international and local Bangladeshi supervisors could work together [26]. Each humanitarian crisis (e.g., forced displacement) has its own nuanced set of challenges that the conventional health system cannot fully capture. In designing the Caring for Carers program, we noted that existing clinical supervision opportunities in Bangladesh did not capture Rohingya voices in terms of the design and delivery of clinical supervision. Given that many MHPSS services are delivered by Bangladeshi practitioners, we wanted to ensure cultural sensitivity in our program by integrating Rohingya perspectives. In this paper, we aim to highlight the different processes of engagement and how the supervision program incorporated voices to ensure that MHPSS supervision practices are culturally appropriate and responsive to the needs of the population.

### The context for Rohingya people in Bangladesh

The Rohingya people have long been persecuted as a religious minority in Myanmar’s Rakhine state, with successive waves of displacement since the 1990s. In 2017, the Rohingya community experienced a mass displacement when more than a million sought refuge in makeshift camps in Cox’s Bazar, the south eastern region of Bangladesh. Exposure to events during the displacement included gunfire, destruction of properties including houses, witnessing dead bodies, physical torture and forced labor, and sexual abuse [27, 28]. In addition, high prevalence of sexual and gender-based violence compounded by inadequate privacy and safe spaces, and limited access to comprehensive psychosocial and mental health support further escalated the challenges experienced in the context. Recently, the overall camp situation has been getting worse with kidnapping, and murders affecting both physical and mental health [29]. These events are reflected in both short and long-term mental health consequences such as posttraumatic stress disorder (PTSD), symptoms of anxiety and depression as well as suicidal ideation [28, 30], all of which are typically seen among people affected by conflicts and displacement [31]. The current mental health care services designed for both displaced communities and MHPSS providers are adversely affected by a number of challenges (e.g., language barriers, understanding and perception of psychopathology, lack of coordination between mental health and psychosocial support services, insufficient clinical supervision, and inadequate human resources to name just a few [30, 32, 33].

## Methodology

### Approach

The Caring for Carers (C4C) Project implemented a 16-month program of group online clinical supervision to MHPSS practitioners in Bangladesh, Tϋrkiye and Syria [34]. This paper focuses specifically on the implementation in Bangladesh. The C4C project adopts a community-based participatory research (CBPR) approach [34], actively involving community members in all stages of the project, from the development of the supervision program to data collection, analysis, and dissemination of the findings. CBPR emphasizes the development of collaborative, reciprocal, and equitable partnerships with community members, viewing them as active and equal contributors to the team rather than mere consultants [35]. This approach can foster trust between service providers and community members, facilitate empowerment, and contribute to reducing health-related inequalities. CBPR underscores the importance of understanding the local context and population directly from a community’s perspective and emphasizes active collaboration [36]. Building on the core principles of CBPR, the C4C project aims to centre the voices and participation of community members, specifically the displaced Rohingya community in Bangladesh, in the development and implementation of the supervision program. In Bangladesh, the program focused on providing clinical supervision to Bangladeshi MHPSS workers, who are the primary providers of MHPSS services in the Rohingya refugee camps.

### Participants and recruitment

Recognizing the need to involve the community directly and gain a deep understanding of cultural aspects, we sought an alternative method to include Rohingya voices through community consultation and an advisory group [37]. A summary of the participants and method of data collection is presented in Table 1, while further details are presented in the following sections.

**Table 1:** Participants profile according to group

Stakeholder groups	No. of Participants	Gender	Method
MHPSS Service users	2	Female	IDI
Community members	5	Male	FGD
Rohingya Advisory Committee	6	2 females and 4 males	Regular Meeting

#### The Rohingya advisory committee (RAC)

Six Rohingya community members were invited to participate in an advisory committee for the duration of the project. We considered the members of the committee as experts and members of the research team, rather than research subjects, so we describe their recruitment separate from participant recruitment. Advisory committees, in general, often rely on official community leaders [15] (Mahji’s) which may prioritise the voices of people holding public positions. We adopted a different approach by selecting members based on existing trusting relationships established by our team over more than a decade of engagement with the Rohingya community in Cox’s Bazaar. We chose to focus on established relationships over trying to ensure representation of all subgroups of the community as we felt this would offer greater honesty and depth to the feedback. As a result, most committee members had arrived prior to 2017. All advisors were over 18 years old, resided within the community, had lived experience of displacement, were oriented to Mental health and Psychosocial Support (MHPSS), and included four males and two females. The committee members were known to the team because of the roles they had played in the community as teachers, journalists, activists, previously elected camp block representatives and researchers. This meant that they were familiar with concerns of a wide range of community members. For example, in a previous research collaboration, some members had recruited community members ranging from adolescent to elderly, including married and unmarried women and men, spiritual leaders and community leaders. They had also participated in programs to document human rights abuses during the 2017 genocide. As a result, of their long experience of working with international organisations, they were all proficient in English.

#### MHPSS service users and Rohingya community members

Two female MHPSS service users from the Rohingya community were purposively sampled for in-depth interviews (IDIs) and five male community members were selected for focus group discussions (FGDs). The recruitment process ensured diversity among participants regarding age, gender, education level, occupation, and mental health orientation.

### Data collection

Gender-segregated methods were adopted to collect data on service experiences and quality from community members and service users. These FGDs were held at the beginning of the project to capture a snapshot of the mental health service use experiences of Rohingya people in the camps. We took a gender sensitive approach for collecting data from male and female participants. One FGD was conducted with five community members at a local Non-Governmental Organization (NGO) where they regularly attended community meetings. In-depth interviews with service users were conducted within the camps where they resided. During the field visit, we discovered that female community members and service users are less accessible than male community members. To align with the cultural norm, gender-specific approach was adopted to collect data. Verbal consent was obtained prior to the FGD and interviews, which were facilitated by the researcher. The process of obtaining verbal consent involved a structured approach developed by the C4C project for conducting stakeholder analysis. It ensured ethical standards and followed basic steps including an introduction from the researcher team, purpose and explanation of the study, details of the participation, confirmation of understanding, consent acquisition, documentation of the provided consent, and follow-up contact information to ensure informed and voluntary participation. The participants’ roles, risks, benefits, and handling of personal information are also informed. Participants provided their consent verbally and the interviewer recorded their consent in the field notes. Participants were asked about their health and mental health, perception of mental health issues within the community, and their understanding of MHPSS provided by various organizations. Both FGD and IDI were done using Rohingya dialect where a Rohingya field volunteer attended as a translator. The Rohingya volunteers were recruited by the project team for data collection from service users and the team thoroughly prepared the volunteers about the purpose and the process of conducting FGD and IDI. Ethical approval for the Caring for Carers study was granted by the University of New South Wales Human Research Ethics Committee (HC210824) and the Department of Clinical Psychology, University of Dhaka (IR211201).

### Data analysis

Data were analysed manually using MS Word and MS Excel to document, summarise and categorise qualitative data from different sources such as, meeting notes from RAC, IDI and FGD transcripts, and reflective discussions with RAC members. The thematic analysis approach was followed as suggested by [38]. The research team used a constructivist approach to data analysis, acknowledging cultural differences in mental health conceptions. They (SJ, OF, RW, GK) conceptualised knowledge and co-constructed it with researchers, RAC, and participants. They employed a latent coding method, considering implied meanings and seeking advice from the Rohingya Advisory Committee. First, all the documents from various sources were reviewed to familiarise us with the content without engaging in the coding process. Secondly, we iteratively generated codes and themes and identified the relationship between the themes. Until finalising the codes and themes, we reflectively evaluated our interpretations and inferences [39]. Findings were presented in a diagram and tabular form to capture major themes. Findings were then presented to the RAC, which provided feedback and assisted in understanding and describing the barriers to participation from their perspective.

### Strategies adopted for engagement

This section outlines the specific and sequential strategies employed to engage community members and integrate the community perspectives in this project from the stakeholder workshops and RAC inputs and activities as an ongoing feedback mechanism.

#### Community engagement through stakeholder consultation

During stakeholder consultations, community members who directly received MHPSS services were included from the camp context. Their perspectives were explored through FGDs and IDIs, providing an initial snapshot of the community’s needs, experiences, and understanding of mental health and well-being.

#### RAC presentations

The RAC members were positioned as experts of their own lived experiences. They conducted a cross-cultural workshop for supervisors at the outset of the Caring for Carers program. This workshop was an integral step in providing training to the people who were going to provide clinical supervision to the Bangaldeshi MHPSS practitioners. The aim was to increase their understanding of Rohingya culture and experience so that they might guide practitioners working in the field to be sensitive to these dynamics. Four advisors presented virtually on the impact of human rights violations on the well-being of Rohingya refugees and emphasized the importance of meaningful participation at the International Society for Health and Human Rights conference in Bogota, Colombia. They shared their experiences as refugees in the camps in Bangladesh, offered perspectives on mental health, and the MHPSS situation in the local context from a local perspective.

#### Ongoing and regular engagement by RAC

RAC members engaged in ongoing meetings to discuss current issues within the camp, reflect on project team feedback and provide cultural insights into ongoing conversations. Meetings were held bi-weekly and facilitated by two international members of the project team who were experienced mental health clinicians to engage members in trauma-informed reflective discussions. This approach fostered meaningful and respectful conversations to explore challenges from their perspectives and to focus on RAC priorities. Sessions were typically structured to invite agenda items from anyone on the group, including concerns about developments in the camp from RAC members or requests for advice on elements of the research project from the international team members. The topics raised were then discussed openly, inviting feedback from all members. When problems with internet connection disrupted the meeting, members would often provide written feedback via Whatsapp. The group was explicitly positioned to leverage the privileges of the international team to platform issues of relevance to the RAC. For example, producing an international article advocating for improved resettlement solutions for Rohingya people [29] and a video featuring community members advocating for community involvement in research [40]. The reflective discussion involved genuine interest in building a trusting collaborative relationship at the outset of the project and leveraging different sources of knowledge (knowledge from lived experience, first-hand experience in providing mental health care in the setting, and academic knowledge) to design a culturally sensitive supervision program.

#### Preparing program materials

A handbook was developed for the supervision program in both Syria-Tϋrkiye and Bangladesh sites. The handbook was initially prepared by an Australian clinical psychologist and supervisor (see Wells, Acarturk [34] for details and a copy of the original handbook in supplementary materials). Rather than specifying a specific model of supervision, the handbook linked to introductory information on the Integrated Model for Supervision provided by the International Federation of Red Cross and Red Crescent Societies [41]. It also encouraged supervisors to work towards supporting practitioners’ development in the areas of human rights, diversity and power; the practitioner and beneficiary relationship; skills and knowledge of MHPSS; case discussion and reflection; therapeutic interventions; and professional, legal and ethical considerations. The handbook included details on considering the co-supervisor (international and local) relationship; group dynamics; session structure; establishing expectations, group rules and provision for practitioner feedback. The handbook was then adapted to the Bangladesh context in collaboration between the Dhaka University team and the RAC through specific meetings in addition to the ongoing meetings. The adapted handbook also included findings from the above IDIs and FGDs, particularly insights from community members who had received services from MHPSS providers.

#### Connection and relationship building between the project and the RAC

Both the RAC and the project team invested in developing a respectful relationship, utilizing ongoing connections through meetings and active contributions to the supervision program. Collaborative efforts were made to reflect on each other’s viewpoints and consider incorporating new content and processes into the supervision program. Continuous engagement through various activities facilitated a longstanding relationship, allowing for genuine feedback on project activities from a cultural perspective [42]. This connection also enabled the RAC to share their current experiences as a displaced community in Bangladesh and discuss barriers and facilitators to incorporating their perspective into a research program. RAC also provided enabling and challenging factors for engaging in the C4C supervision project.

## Results

Findings integrated community perspectives and experiences, highlighting six major themes derived from stakeholder workshops with Rohingya community members and service users. In addition, the results focus on demonstrating how insights from the RAC helped to shape our understanding and analytical approach.

### Cultural understanding of displacement

Community members and service users discussed post-displacement trauma, distress, and worry, particularly regarding the 2017 mass displacement. However, RAC moved the spotlight to the experiences of displacement that happened decades ago and the current situation in the camp context. It helped us to see the displacement according to time context and differences in experiences among communities who had been recently displaced and those who were displaced a long time ago. This helped to contextualize current vs. prior displacement events. While the Rohingya community endured significant hardship, including limited access to basic needs, the community also demonstrated resilience and a strong sense of hope. They emphasized the importance of preserving their cultural heritage amidst ongoing difficulties.

### Mental health needs and perception of MHPSS among the Rohingya community

**Fig. 1:**
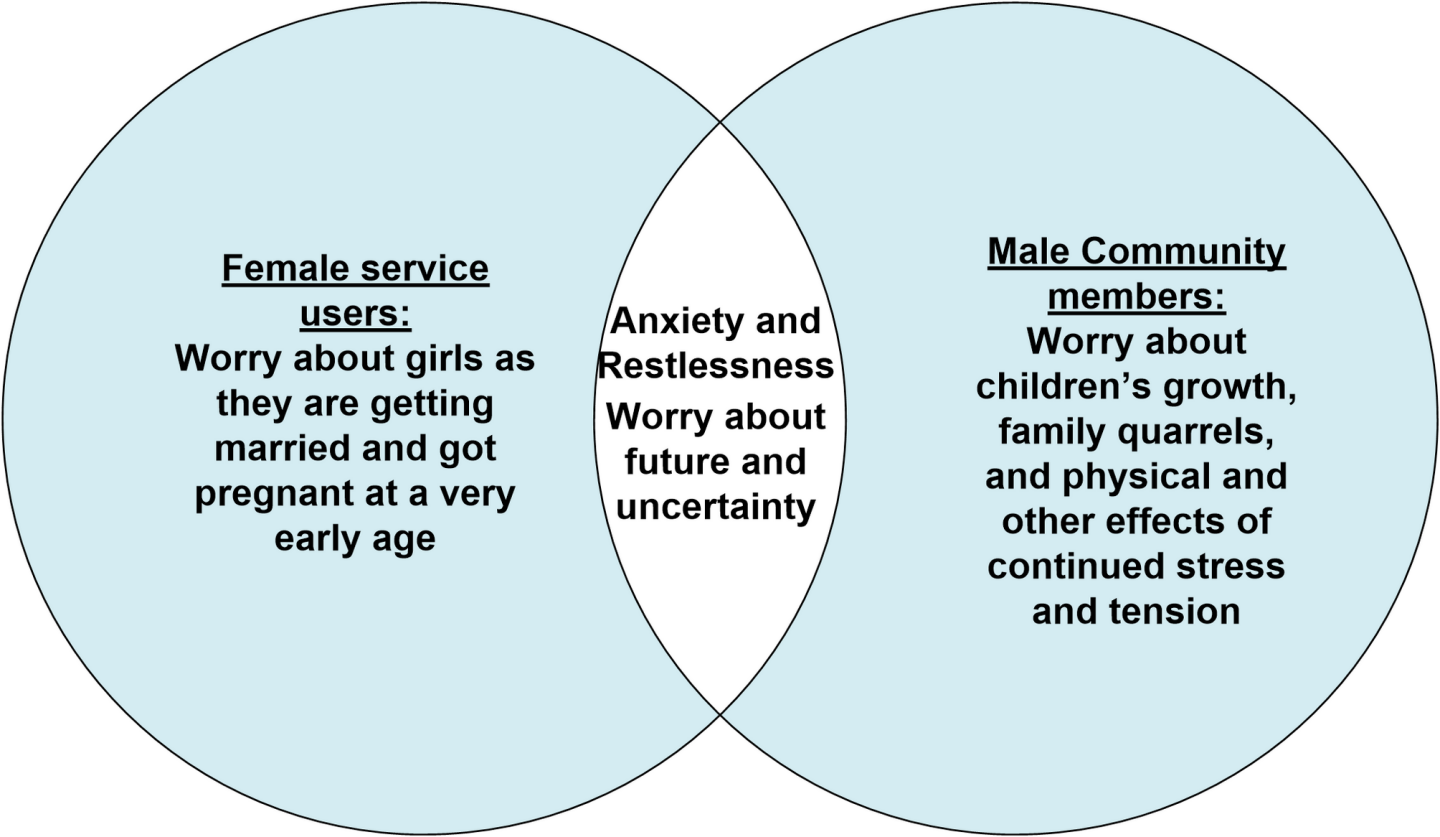
Conceptualizing mental health status according to gender

Stakeholder workshops enabled community members and service users to articulate their mental health needs and perceptions on the MHPSS services. Community members described a severe sense of restlessness and anxiety, exacerbated by environmental stressors such as hot weather, overcrowded living conditions, and uncertainty about their future. Specific concerns, such as family conflicts, domestic violence, and early marriage of young girls, were also highlighted and mentioned by the female services users also. Female service users expressed appreciation for MHPSS intervention, particularly in learning breathing exercises and communication strategies to manage household conflicts and stress. However, male community members viewed these interventions as temporary solutions, expressing that their ultimate need was to return to their homeland (Fig. 1). The RAC helped further contextualise these findings, validating the deep-seated anxiety and insecurity within the community.

### Appreciation and curiosity about counselling and supervision process

Over time, RAC members expressed growing curiosity and appreciation for the counselling and supervision processes of the C4C program. They raised critical questions about the linguistic and cultural background of MHPSS service providers, recognizing that language barriers could hinder the expression of deeper emotions, such as who are the counselors, are they from Chattogram (a region located near the camp setting has similar dialects with Rohingya community). The RAC’s affirmation of the intervention’s content—focused on human rights, diversity, and the relationship between practitioners and beneficiaries—enabled the project team to adapt and customize program materials to reflect what was important to the Rohingya community.

### Situational understanding

RAC members provided valuable situational insights regarding the camp environment, services, and facilities in Ukhya, Teknaf, and adjacent areas where the Rohingya reside. Their input during reflective discussions contributed to understanding the day-to-day challenges faced by refugees, including the impact of natural disasters such as cyclones and the treatment of community members by local staff. These insights were crucial in shaping the scope of the supervision program and ensuring it addressed the specific needs of the community.

### Experiential information and historical background

The RAC played an essential role in sharing lived experiences and the historical context of the Rohingya community. Their narratives provided the project team and Bangladeshi MHPSS providers with a firsthand understanding of the oppression and torture experienced by the Rohingya. The committee also contributed historical and ancestral information to the supervision handbook, helping supervisors contextualize mental health determinants from the pre-displacement to post-displacement phases. While this experiential knowledge enriched the program, it remains unclear how participation in the project impacted RAC members, who continue to live under the same precarious conditions.

### Barriers to involvement

Despite the investment and enthusiasm of all members, socio-political factors emerged as the main determinants of the scope and longevity of our collaboration. After co-developing the supervision program, security concerns necessitated less direct forms of input during implementation of the supervision. In response to this, the RAC continued to provide feedback and consultancy about implementation-related challenges while we worked together to platform socio-political issues in international forums.

## Discussion

The present paper endeavored to demonstrate the steps and processes of the inclusion of Rohingya community members into the development and implementation of the clinical supervision program for Bangladeshi mental health workers providing MHPSS to displaced Rohingya people. The inclusion of the RAC helped the supervision program support Bangladeshi MHPSS professionals to consider the culture and context-specific needs of the displaced Rohingya community in Bangladesh. The RAC played a prominent role in providing expertise about Rohingya culture, mental health problems among Rohingya people, provision of an inclusive culturally sensitive model of mental health care, and skills and knowledge targeted during supervision. Based on learnings from our ongoing relationship with the RAC, we propose a “PEACE” Model to delineate the key steps and components of ensuring the meaningful integration of the community voice in humanitarian mental health research. PEACE stands for Participation; Expertise; Agency; Connection and Empowerment. Below, we describe how we endeavoured to build PEACE model through our collaborative engagements despite the challenges faced during implementation. Table 2 describes the ways that PEACE might be realized in a LMIC context, and considers the barriers and enablers. We then describe the theoretical bases for each of the elements of the model.

**Table 2:** PEACE model describing components of the model, strategies used in our project and reflection on the barriers and enablers in humanitarian contexts

Component	Description	Strategies used in C4C	Barriers in the Humanitarian context	Enablers in the Humanitarian context
**Participation**	Participation aspires to establish a collaborative relationship based on a mutual understanding toward a common goal and equal contribution.	Ongoing meetings with RAC throughout the project	In general practice participation activities are often focused around priorities of NGOs or researchers. Community voices risk being seen as formalities, rather than central to the process	Many NGO vision and mission statements incorporate participation as important, including models/frameworks promoting the inclusion of all stakeholders
**Expertise**	Marginalised community members are experts with lived experiences and cultural knowledge that is as important as scientific knowledge, if not more so	Community FGDs and IDIs at start to inform program design. Supporting RAC to develop their own materials on their own terms for presentations, conferences and handbooks	Traditional academic and development settings prioritize expertise based on hierarchy, which can reduce curiosity to genuinely listen to the community. Lack of regulatory systems in LIMCs might affect level and nature of community engagement.	Emphasizing context-specific expertise; treating community inputs as valuable; recognizing that issues raised are important to the community
**Agency**	Forming trusting, equitable partnerships in knowledge production fosters community members’ sense of efficacy and agency over the matters that affect their lives.	Inviting the RAC to determine priorities for international knowledge translation activities	Community researchers and health workers are often asked to implement projects without being given any decision-making powers. Agency promoting approaches require additional resources.	Encouraging a more inclusive decision-making approach, reflecting community input in project implementation, and promoting democratic processes that mobilize local resources.
**Connection**	Trust can only build through relationships founded on genuine care and respect in both directions	Regular meetings with a trauma informed approach that responded to the emotional, social and political concerns of the group	Historical oppression and violence erode trust. Speaking openly might pose risks if others report to authorities or armed groups.	Trust can be easier to establish with external parties (e.g., foreigners) outside the local power dynamics.
**Empowerment**	Empowerment can be an outcome of a collaborative participation process	Including experts in program and public forums	Despite long involvement from international NGOs and researchers, little change is perceived by the community. This may lead to discouragement.	Community members are willing to invest their time and knowledge to improve their situation. Donors and programs can prioritize enhancing community skills

### PEACE model of integrating voices of marginalized communities in LMICs

**P**articipation: Active participation of Rohingya community members in the design process of the supervision program was achieved through several meetings and workshops with the research team and supervisors.

**E**xpertise- Drawing from the main principles of CBPR, the C4C project positioned Rohingya community members as experts with lived experience, considering their living knowledge as equally important as scientific knowledge, if not more so.

**A**gency- In our project, we aimed to support the agency through mutual learning and exchange activities. For example, the RAC members taught the supervisors about the displacement-related stressors and mental health needs of the Rohingya people and collaborated on the content of the supervision. The international team supported the RAC to platform their concerns on an international stage.

**C**onnection- Strengthening ethical and quality delivery of mental health care for Rohingya people in Bangladesh as a higher-order goal. Our different legal, social and cultural positions meant that there were many challenges in our work. Genuine connections and trust supported our ability to keep working together despite these barriers.

**E**mpowerment- Although our collaborative relationship helped the RAC take ownership of their part and contribution in designing supervision program, we recognized that empowerment as a desired outcome, hinges on several, mostly structural, enabling factors such as policies allowing official employment of Rohingya people as research partners and official recognition of their presence in Bangladesh. Please see Table 1 for a summary of the model components and barriers and enablers in humanitarian contexts raised by the RAC. Below we discuss each element of the model with reference to relevant literature.

**P**articipation: Participation is at the core of a community-based participatory research approach which prioritizes the involvement of community members at each stage of the research process [43]. Participation of community members promises an opportunity to vocalize needs and preferences and share local knowledge, though does not guarantee the realization of this promise [44]. Given the intricate histories of discrimination and exclusion, people from disadvantaged backgrounds (e.g., forcibly displaced people) might be hesitant to participate and share information. Participation based on trust and mutual understanding is a prerequisite to form a collaborative relationship through which community members can be meaningfully involved in multiple stages of research [45]. In our project, we worked toward forming a trusting collaborative relationship with the Rohingya Advisory Committee (RAC). Our personal relationships and previous working history with some committee members helped us scaffold a solid foundation for collaborative participation and recruiting other members. A positive start in the early stages of the relationship formation provided us with enormous advantages and saved us time. Yet, we were cognizant that it would never guarantee continuation of trust in later stages of collaboration. Considering our diverse social identities and group memberships, including Rohingya community members, local researchers, and international researchers, hierarchical power dynamics among team members influenced all our undertakings. This necessitated us to continuously reflect on our positionality and power dynamics and re-evaluate when and how we could trust each other. In an ideal collaborative relationship, the involvement of community members should span all stages of research, from inception through implementation to data analysis and dissemination. Given the ways in which local socio-political and security issues influenced the RAC involvement, we argue that to realise the full potential of collaborative participatory research, a detailed assessment of socio-political and cultural factors and strategic planning for potential barriers to participation should be conducted [15]. Our project upholds active participation of the community members, service users, and RAC rather than taking an instrumental step [12].

**E**xpertise- It has been a long tradition in psychiatry and related disciplines that people from disadvantaged backgrounds have been subject to systematic discrimination in all forms of knowledge production activities. Until recently, the ownership of knowledge was assumed to be in the hands of those in privileged positions, such as academics [46]. The accruing evidence on the benefits of participatory research has given momentum to inclusion of people with lived experiences in research as equitable partners [47, 48]. Equitable distribution of power of knowledge among researchers and community members entails adapting a critical decolonial lens through which community members can bring extensive historical, cultural, and practical knowledge about the subject matter to the table. Decolonization of the power of knowledge signifies a paradigm shift in global health research praxis, involving redistribution of power and equitable inclusion of diverse forms of knowledge, such as local and indigenous knowledge, in knowledge production [17, 49]. Decolonizing power of knowledge can be daunting for researchers and traditional owners of knowledge because the process pushes researchers to think out of box, a task often hindered by the Western-imposed colonial ways of conducting research. It takes more than acknowledging the inherent power hierarchies among team members. Similar to the experiences of other researchers working towards decolonizing knowledge [50], our collective process has taught us that it requires openness, genuine interest, curiosity toward local knowledge, and flexibility on the part of researchers to challenge power hierarchies. Beyond individual attitudes, it is also important to acknowledge that limited time and resources in research projects can pose major impediments to achieving this goal. Attention to local context, trusting collaborative relationship, and appreciative listening to community voices, on the other hand, are the enablers of breaking the vicious cycle of power hierarchies around knowledge and its production.

**A**gency- Forming relationship, and equitable partnership in knowledge production fosters community members’ sense of efficacy and agency over the matters that affect their lives. The mental health field has long suffered from embracing a deficit-based approach and perceiving community members as passive recipients of mental health care. This approach led to the labeling and stigmatization of community members with lived experiences of mental health issues. In contrast, a strengths-based approach centers on individuals’ agency in mental health care, helping them regain a sense of control and influence over factors affecting their lives [13]. Fostering a sense of agency in knowledge production can have far-reaching health and social benefits for community members via several mechanisms such as increasing motivation to engage in further research projects and engaging in advocacy and collective action for systemic change [45]. By prioritizing community voice and collaboration, community-based participatory research provides a platform for community members to exercise and restore their sense of control and agency. It also maximizes the relevance and fit of mental health interventions for targeted communities [51]. We note that such processes can only support agency within the structure of legal and political rights that are available to displaced communities in the local context. A research team’s desire to promote agency should be balanced with consideration of the real dangers that displaced community members may face in speaking out. Community members must be afforded legal and social protection to safeguard their rights and prevent exploitation and discrimination.

**C**onnection- CBPR also serves as a bridge between researchers and community members and fosters connections between these traditionally siloed groups. Based on the Intergroup Contact Theory, frequent positive contact between members of different social groups, researchers, and community members in this case, can facilitate connection between people and enable recategorization of group memberships more inclusively [52]. Inclusive group identification such as perceiving both community members and researchers as experts can enhance positive attitudes and behaviors towards each other. This also helps promote common goals for collaboration [53]. Whether contact experiences between different group members is positive or not hinges on some foundational elements such as trust and mutual understanding. In our project, our existing trusting relationships helped us to work together on some contextual obstacles. However, maintenance of positive changes related to connection for a longer period is subject to investigation.

**E**mpowerment- In our model, we consider empowerment as the outcome of the collaborative participation process. There can be several forms of empowerment such as relational empowerment and capacity-building [54]. Relational empowerment refers to the equitable sharing of power in the relationship which is achieved through developing a shared identity and goal in the relationship [55]. On the other hand, capacity-building includes both skills and knowledge development through collaborative learning and mobilizing resources, thereby enhancing the sustainability of community-based research [54]. Having acknowledged the contextual difficulties and obstacles in a given setting, this may not be always achieved following the collaborative research process. Empowering Rohingya refugees to actively participate in decision-making processes that affect their lives is essential for promoting their agency and dignity. Sustainable education and livelihood opportunities for Rohingya refugees is crucial for promoting self-reliance and economic empowerment.

“You can’t have peace until everybody is equal” Kwame Ture [56].

Finally, we would like to reflect on our hope for peace for the Rohingya people, who have been through genocide and years of displacement. The story of Rohingya refugees in Bangladesh is one marked by resilience, courage, and the unwavering pursuit of dignity and justice. As Rohingya people find themselves in a land that is not their own, they continue to support each other, preserve cultural heritage, and seek education, to plant seeds of hope for a better future, one where freedom and opportunity are not distant dreams but tangible realities. Our attempts to work together through the PEACE model proposed above are aimed ultimately at peacebuilding. However, this cannot overcome the real-world exclusions and human rights abuses that many in Rohingya and Bangladeshi communities experience. Sovereignty and self-determination are required to make peace a sustained reality.

## Conclusion

This paper proposes a framework for community engagement (i.e., PEACE) in LMICs developed through collaboration with an HIC country, an LIMC and the community from the displaced context. The PEACE model aims to invite community voices at each step of program development and implementation. This framework provided a pathway to incorporate community perspectives into the conceptualization and development of a clinical supervision program. The importance of this framework lies in its emphasis on community engagement in the context of LMICs. By incorporating the voices and perspectives of the local community, particularly those in displaced contexts, the framework ensures that program development is more inclusive, culturally relevant, and responsive to the actual needs of the population it aims to serve. This participatory approach can enhance the effectiveness, acceptance, and sustainability of interventions, particularly in clinical supervision programs, by ensuring that community members play an active role in shaping the solutions that impact their lives.

## Electronic supplementary material

Below is the link to the electronic supplementary material.

Supplementary Material 1

## Data Availability

The datasets analysed during the current study are not publicly available as due to being qualitative data from a small number of participants that contains sensitive personal information about mental health, but are available from the corresponding author on reasonable request.
